# Comprehensive anaesthesia management strategies for orthognathic surgical procedure

**DOI:** 10.4317/medoral.26833

**Published:** 2024-10-13

**Authors:** Blanca Tapia-Salinas, Iñigo Aragón-Niño, José Luis Del-Castillo-Pardo-de-Vera, José Luis Cebrián-Carretero

**Affiliations:** 1 Anesthesiology and Reanimation Department La Paz University Hospital, Madrid, Spain; 2 Oral and Maxillofacial Surgery Department La Paz University Hospital, Madrid, Spain

## Abstract

**Background:**

Orthognathic surgery is commonly performed to correct malocclusion and facial asymmetry, typically in young and healthy patients. However, it's crucial to recognize that facial deformities can also be features of various syndromes, complicating the surgical and anesthetic management.

**Material and Methods:**

This review examines the key factors influencing anaesthesia management in orthognathic surgery patients. The perioperative care process was analyzed, focusing on the surgical procedure, airway management, and anaesthesia techniques. Additional factors considered include bleeding control, postoperative nausea and vomiting (PONV), antibiotic prophylaxis, analgesia, and deep venous thrombosis (DVT) prevention.

**Results:**

The review highlighted the critical role of comprehensive anaesthesia management in determining the outcomes of orthognathic surgery. Proper airway management, effective bleeding control, and the prevention of PONV and DVT were identified as significant factors in optimizing patient recovery. The use of protocols and a multidisciplinary approach were emphasized as essential in minimizing complications and improving postoperative outcomes.

**Conclusions:**

Comprehensive and carefully planned anesthesia management is vital for the success of orthognathic surgery. Employing a multidisciplinary approach and adhering to established protocols can enhance patient outcomes and expedite recovery, especially in cases involving complex syndromic patients.

** Key words:**Anaesthesia management, orthognathic surgery, malocclusion.

## Introduction

Orthognathic surgery is performed to correct malocclusion and facial asymmetry. Although it usually involves young healthy patients, it is important to bear in mind that facial deformities are sometimes a characteristic of many different syndromes.

The aim of this paper is to analyze the main factors involved in the anaesthesia management of these patients.

Orthognathic surgery is a surgical procedure that restores a patient's entire facial structure by repositioning the maxillary and mandibular bones to address dentofacial abnormalities. Due to its complexity, precision and planning are essential components of this surgery and are also key factors in the approach to anaesthesia management both overall and at specific stages of the procedure.

- Surgical procedure

Orthognathic surgery is a surgical intervention that corrects dentofacial deformities by moving the maxillary and mandibular bones to balance the facial characteristics of the patient.

The procedure, known as bimaxillary orthognathic surgery, involves both maxillary and mandibular osteotomies that are performed either independently or simultaneously.

- Maxillary surgery

Maxillary orthognathic surgery is performed to realign the maxilla in order to achieve facial harmony and restore the functionality of the bone, which is essential for chewing, breathing, and speaking.

Orthognathic surgery of the maxilla consists of cutting the maxillary bone in a procedure called a Le Fort I Osteotomy, in which the maxillary bone is advanced, retruded, lengthened, shortened, or rotated.

- Mandibular surgery

The most commonly performed orthognathic surgery on the mandible is mandibular advancement surgery. This procedure is necessary for individuals with a small, receding jaw in relation to the maxilla - a condition known as retrognathia or class II occlusion. In individuals with a protruding jaw, a procedure called mandibular setback is indicated.

- Bimaxillary surgery

In most orthognathic surgery patients, both the maxilla and mandible must be repositioned to achieve correct occlusion and facial harmony in a procedure known as maxillomandibular surgery, or bimaxillary orthognathic surgery.

Once the maxillary and mandibular bones are in the desired position, they are fixed in place with titanium plates.

Being a particularly complex procedure, precision and planning are key factors not only in the surgical technique itself, but also in the approach to anaesthesia management both overall and at specific stages of the procedure.

## Material and Methods

We perform a systematic review of the literature regarding anesthesia management in orthognathic surgery. The review process involved a search in PUBMED for relevant articles, using keywords related to "anesthesia management", "orthognathic surgery", "perioperative care" and other related terms. The search included studies published until the current year.

Inclusion criteria were defined to focus on key aspects of anesthesia management specifically for orthognathic surgery, including airway management, bleeding control, postoperative nausea and vomiting (PONV) prevention, antibiotic prophylaxis, analgesia, and deep venous thrombosis (DVT) prevention. Exclusion criteria were also established to omit studies that did not directly relate to the perioperative anesthesia management or those involving unrelated surgical procedures.

Full texts of the selected articles were then thoroughly reviewed. Data extraction was performed to gather information on the methodologies, findings, and conclusions related to anesthesia management in the context of orthognathic surgery.

The analysis aimed to identify common strategies and techniques, assess their effectiveness, and highlight areas where further research is needed. The systematic approach ensured a comprehensive understanding of the current best practices in anesthesia management for patients undergoing orthognathic surgery.

## Results

After the systematic review of the included articles, we found the following points to be of relevant interest, in order to define orthognathic surgery perioperative management and recommendations

1. Airway

2. Intubation

3. Positioning, care of pressure sores and eye protection

4. Throat pack

5. Fluid management, oedema protection

6. Bleeding

7. Postoperative nausea and vomiting

8. Intra and postoperative analgesia

9. Antibiotic prophylaxis

10. DVT prophylaxis

11. Emergence and extubation

- Airway

Airway management is straightforward in most patients; however, direct laryngoscopy can be difficult in patients with retrognathia, maxillary protrusion, or limited mouth opening (1,2). Videolaryngoscopy (VL) facilitates vocal cord visualization. Intubation is faster with the McGrath videolaryngoscope (Aircraft Medical, Edinburgh. UK) compared with Macintosh or GlideScope (Verathon, Inc, Bothell, WA) laryngoscopy in patients with an anticipated difficult airway (3), and has also been shown to increase the success rate in patients with a simulated difficult airway (4). The McGrath VL also reduces the need for Magill forceps, probably because the airway does not need to be as carefully aligned as in conventional laryngoscopy, and VL provides a more direct route from the nasopharynx to the trachea, particularly in patients with an anterior larynx, thereby reducing the need for manipulation of the nasotracheal tube.

- Intubation

In maxillofacial surgery, intubation is performed through the nasal cavity. The tracheal tube is taped to the forehead to give a clear view of the face and facial features and allow the surgeon to correctly align the mandible and maxilla.

Prior to insertion of the tracheal tube, the nasal cavity must be prepared with vasoconstrictors, such as oxymetazoline. Nasal intubation is not suiTable for long procedures, because suctioning and ventilation are difficult through the tube owing to its small calibre, shape and length.

- Eye protection and perioperative pressure injuries

Failure to close the patient's eyes can result in corneal ulceration, and pressure from surgical instruments or the surgeon’s fingers can cause corneal abrasion.

To prevent these adverse events, the eyes must be lubricated and covered with eye pads.

Patients undergoing orthognathic surgery are susceptible to pressure injuries due to the length of the procedure, so it is essential to take preventive measures. Even though young, healthy patients with no vascular pathology or diabetes are less likely to develop pressure injuries, it is advisable to protect the skin over bony prominences, especially in patients who are thin or underweight (5). The most common sites for pressure injury are the sacrum and the heel of the foot.

- Throat packs

Throat packs are commonly inserted by an anaesthetist or surgeon after induction of anaesthesia for dental, maxillofacial, nasal, or upper airway surgery.

The purpose is to absorb blood and other secretions, debris, and tooth fragments in order to keep the airway clear before extubation.

All team members must be aware of the positioning of the gauze in the pharynx (6).

**Figure 1:**
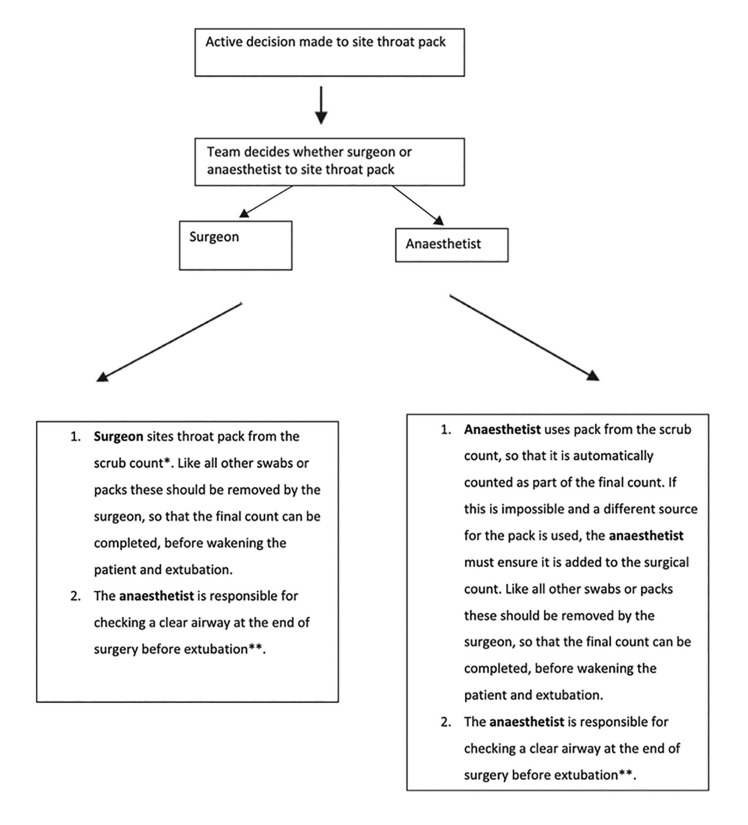
Protocol for throat pack use.

Some authors have put forward strategies to ensure that the pack is removed at the end of the surgical procedure. These include tying or suturing the pack to the endotracheal tube (not possible with nasotracheal tubes), leaving a portion of the pack protruding from the mouth, placing reminder labels, using a checklist, etc. The risk of pack retention is particularly high when there is a change of anaesthetist or other team member, so care must be taken in these circumstances. Some authors recommend performing direct laryngoscopy and/or suctioning the pharynx before extubation to confirm that the pack has been removed. In 2018, the Difficult Airway Society (DAS), the British Association of Oral and Maxillofacial Surgery (BAOMS), and the British Association of Otorhinolaryngology, Head and Neck Surgery (ENT-UK) published an evidence-based consensus statement containing a protocol for throat pack use. (6) (Fig. 1).

- Fluid therapy and oedema management.

Intraoperative fluid therapy is an essential part of anaesthesia, but it is important to bear in mind that the fluids used are not only a vehicle for drug administration, but are also drugs themselves.

Conventionally, high volumes of crystalloids have been administered to patients undergoing major surgery to compensate for perioperative dehydration and intraoperative losses due to fluid shift into the “third space”. However, there is evidence that a positive fluid balance is associated with complications and even with an increased risk of mortality.

Physiological fluid flow does not cause interstitial oedema; however, damage to the vascular endothelial barrier will result in a pathological fluid flow that produces oedema at the surgical site. The endothelial surface layer is made up of endothelial cells and the endothelial glycocalyx layer (composed of membrane-bound glycoproteins proteoglycans and glycosaminoglycans), which preserves transendothelial permeability, regulates inflammation, and prevents platelet aggregation and leucocyte adhesion. All these functions are lost in the event of fluid overload.

Evidence has shown that patients are not usually hypovolemic after preoperative overnight fasting, and that even after prolonged preoperative fasting, cardiopulmonary healthy patients remain intravascularly normovolaemic.

More liberal administration of crystalloids in healthy patients undergoing moderate-risk surgery leads to a better recovery profile compared with patients who received restricted amounts of the same crystalloids.

Perioperative protection of the endothelial glycocalyx is a plausible strategy for the prevention of interstitial oedema (7).

It is interesting to note that 30% of every litre of 0.9 % saline administered remains in the intravascular compartment after equilibration. Colloid administration is context sensitive, but when used in normovolaemic patients, 79% of gelatines and a 84% of hydroxyethyl starch remain in the intravascular compartment.

Most fluid therapy studies have been performed in abdominal surgery, but an interesting paper by Nishimura *et al*. reported that infused fluid often moves from the intravascular to the interstitial space. The authors also observed that increases in infused fluid volume may increase intravascular pressure, leading to more outward fluid movement from the intravascular to the interstitial compartment, suggesting that this could contribute to postoperative oedema , particularly in the presence of anaesthesia and/or surgical stress (8).

Fluid therapy also has an impact on hospital length of stay (LOS).

Huamán *et al*. (9) found that the administration of intravenous fluid was significantly associated with increased LOS, and that

colloids, specifically hydroxyethyl starches, were significantly more likely to increase LOS compared with crystalloids.

- Oedema.

Several methods are more effective than restrictive fluid therapy in reducing postoperative oedema.

1. Cooling is highly effective against oedema and has no side effects. For this reason, intraoperative irrigation fluids are cooled and hialotherapy is used after surgery (10).

2. Anti-Trendelenburg positioning (at least 45º) could help prevent postoperative oedema and inflammation by reducing head and neck congestion.

3. The administration of corticosteroids is thought to inhibit mast cell production and secretion of cytokine, kinin and histamine. They also reduce the production of thromboxane and bradykinin, thus reducing blood vessel dilatation and permeability (11). Glucocorticoids, especially dexamethasone (> 0.15 mg/kg)

not only shorten LOS, but also reduce postoperative oedema (11). Dexamethasone also has other advantages: it has an analgesic effect; it is thought to inhibit prostaglandin synthesis (12); it protects against postoperative nausea and vomiting; and contributes to nerve healing after injury to the dental alveolar nerve (13).

High-dose dexamethasone (> 0.2 mg/kg) has opioid-sparing effects and also decreased pain scores, and for this reason it is used in orthognathic patients (14). No side effect have been registered in our patients

- Bleeding.

The maxillomandibular region is highly vascularized and intraoperative blood loss is often significant, despite controlled hypotension during surgery (15). Although, according to the literature, 27% to 30% of patients undergoing bimaxillary osteotomy procedures require allogenic blood transfusions, none of our patients have so far required transfusion; however, haemostasis improves the view of the surgical field, and hence significantly reduces operating time.

Hypotensive anaesthesia (lowering the patient´s blood pressure during anaesthesia) is effective in reducing surgical bleeding, and has been practiced for several decades (16).

In hypotensive anaesthesia, baseline mean arterial pressure (MAP) is reduced by around 30%, although this percentage will vary depending on the patient’s pathology (17,18).

Optimal analgesia is the key to blood pressure management, but other pharmacological strategies may also be required. Fentanyl and remifentanil are both used to achieve pain control, and perfusion of dexmedetomidine (an alpha 2 agonist) has recently been added to boost analgesia and lower blood pressure.

The two main strategies for achieving hypotensive anaesthesia are:

1. Deep anaesthesia

2. Standard anaesthesia and administration of hypotensive drugs

Blood pressure can be reduced with several different drugs. The ideal hypotensive drug should be easy to administer, have a short time of onset, an effect that disappears rapidly when administration is discontinued, rapid elimination, and no side effects.

3- Nitrates, though popular in the past, are now no longer used because of their side effects.

4. Beta-adrenergic receptor antagonists act by reducing cardiac output. Esmolol, which is metabolized by plasmatic esterase, is the drug of choice due to its rapid onset and short duration.

5. The calcium channel blocker clevidipine has favourable pharmacokinetic characteristics and is effective in reducing blood pressure.

Tranexamic acid (trans-4-(aminomethyl) cyclohexane carboxylic acid) (TA) is a synthetic derivate of the amino acid lysine that competitively inhibits the activation of plasminogen to plasmin by binding to Kringle domains. Tranexamic acid is also a competitive inhibitor of tissue plasminogen activator. It blocks the lysine binding sites of plasminogen, resulting in inhibition of plasminogen and fibrin binding to plasminogen, and therefore impairment of fibrinolysis. It is distributed throughout the body and has a plasma half-life of 120 min. The best-known trial, Clinical randomisation of antifibrinolytics in significant haemorrhage (CRASH-2), assessed the effects of early administration of tranexamic acid in trauma patients with, or at risk of, substantial bleeding, and showed that administration of TA reduced bleeding-related mortality (14.5% vs 16%; relative risk (RR) 0.91, 95% CI 0.85-0.97; *p*=0.0035), but did not reduce transfusion requirements (19).

Though the efficacy and safety of the drug have been established, there is no consensus about the ideal time of administration and the dose (perfusion, bolus), especially in orthognathic surgery. However, there is evidence that 10 mg/l is required for 80% inhibition of tissue activator activity, so this is the dosing regimen used in our department.

TA is contraindicated in patients with kidney failure, thromboembolic diseases, haematuria, and in pregnant women.

Dakir *et al*. confirmed that preoperative administration of an intravenous 10 mg/kg bolus of tranexamic acid reduced blood loss compared with placebo during surgery (19).

- Postoperative nausea and vomiting.

Postoperative nausea and vomiting (PONV) is a common adverse effect of anaesthesia and surgery, and occurs in up of 80% of high-risk patients.

Nausea and vomiting are not only highly distressing, but are also associated with a longer stay in the post-anaesthesia care unit (PACU), increased health care costs and hospital re-admission (20).

Patient-specific risk factors for PONV in adults include female sex, a history of PONV and /or motion sickness, non-smoking status, and young age (21).

Previously, patients considered a low risk for PONV were given either no prophylaxis or only 1 prophylactic drug. This approach has now changed considerably because PONV risk scores only provide a rough estimate, and patients identified as low risk may still develop PONV. Furthermore, PONV scores do not take into account factors such as the emetogenic risk of the surgical procedure and inter-individual variability in antiemetic effectiveness (22).

As mentioned above, certain types of surgery, including orthognathic procedures, may be associated with an increased risk of PONV.

Several drugs with different mechanisms of action are available: corticosteroids (dexamethasone, methylprednisolone), antihistamines (dimenhydrinate, promethazine) and anticholinergics (scopolamine), neurokinin1 receptors (aprepitant, fosaprepitant, etc.), dopamine- 2 receptors (amisulpiride, droperidol, haloperidol, etc), and 5-hydroxitryptamine3 antagonists (palosetron, dolasetron, granisetron, ondansetron, etc.) (23,24), (Table 1).

If general anaesthesia is required in orthognathic surgery, the use of total intravenous anaesthesia (TIVA) has been shown to reduce PONV. A meta-analysis of 229 randomized controlled trials in different surgical procedures concluded that TIVA offers a benefit in reducing the incidence of PONV compared with volatile anaesthesia (25). The use of propofol for anaesthesia (or sedation) is associated with a 3.5-fold reduction in the incidence of PONV in adults and a 5.7-fold reduction in children (26).

Sub-hypnotic doses of propofol (20-40 mg) are also effective as a rescue treatment for PONV.

Other strategies that could minimize PONV risk are nitrous oxide-sparing anaesthesia, reversal of neuromuscular blockade with sugammadex, and use of intravenous lidocaine and dexmedetomidine infusion for analgesia (due to the opioid-sparing effect of these drugs) (27,28). Opioid-free anaesthesia reduces PONV risk, but this benefit must be weighed up against the risk of inadequate analgesia, hypertension, and bleeding.

Intraoperative fluid administration may affect the risk of PONV. For example, 10-30 ml/kg infusion of intraoperative crystalloids reduces the risk of PONV; however, crystalloids should be avoided in orthognathic surgery (29).

The combination of ondansetron and dexamethasone is one of the most widely studied and utilized multimodal PONV prophylaxes. Many new drugs are being added to this combination, with promising results.

Palosetron: this second generation 5-HT3 receptor antagonist has a 100-fold affinity to the 5-HT3 receptor and a terminal half-life of 40 h. PONV is significantly reduced for 72 hours after surgery, and it is usually used in high-risk female patients. Monotherapy for PONV prophylaxis is more effective than other

5-HT3 antagonists and dexamethasone, and its efficacy is comparable to aprepitant (30).

Its main drawback, however, is its high cost.

Aprepitant: This competitive neurokinin (NK-1) receptor antagonist was initially approved for the treatment of chemotherapy-induced nausea and vomiting,

and is administered orally for this indication. However, a prodrug of aprepitant, fosaprepitant, with a half-life of 9-13 h and a duration of action of 40 h, is available for intravenous administration . Fosaprepitant is more effective in preventing PONV than ondansetron, and has the same efficacy as palosetron (31).

Amisulpiride: Dopamine receptor antagonist and antipsychotic approved for prophylactic and rescue therapy of PONV. It is administered intravenously at a dose of 5-10 mg.

A wide variety of nonpharmacologic techniques have been used to control emetic symptoms in the postoperative period, such as acupressure, acupuncture and transcutaneous electrical nerve stimulation.

Alcohol pads applied under the nose are a highly cost-effective treatment for transient PONV in adults and children.

There is no reliable evidence that aromatherapy reduces postoperative nausea and vomiting (32).

Some authors recommend P6 acupoint stimulation for PONV prevention. This has no side effects and significantly reduces nausea, vomiting and the need for rescue antiemetic drugs.

Aside from pharmacological strategies, it is important to bear in mind the role of throat packs and stomach aspiration prior to emergency surgery in reducing the risk of PONV.

- Analgesia.

Many head and neck procedures are not associated with severe postoperative pain,

and pain management with non-steroidal anti-inflammatory drugs and paracetamol usually suffices.

However, evidence shows that up to 21% of orthognathic patients continue to feel pain 1 year after surgery (33). Severe, acute, postoperative pain increases the risk of the pain becoming chronic (34). Multimodal analgesia is essential, and local anaesthetic infiltration by surgeons must be combined with opioids to decrease side effects and improve their adjuvant effect (35).

Some authors recommend pre-emptive analgesia, arguing that postoperative pain can be prevented if certain analgesics are administered before the surgical stimulus; however, both peripheral and central stimuli must be blocked in order to achieve this goal. In 1983, Woolf defined pre-emptive analgesia as the treatment of pain before the surgical stimulus and the maintenance of this treatment during the high intensity harmful stimuli and during the postoperative period. Kissin suggested changing pre-emptive to preventive analgesia in order to limit the technique to the pre-operative period (36).

In our practice, we administer NSAIDs (dexketoprofen 50 mg), acetaminophen 1 g, and dexamethasone 0.15 mg/kg immediately after induction to reduce the hypersensitisation of pain receptors, and then administer a further dose immediately after the surgery or immediately before admission to the PACU.

Dexmedetomidine is an α2-adrenoreceptor agonist that primarily inhibits norepinephrine release and attenuates central nervous system excitation. The binding of postsynaptic receptors by α2-agonists leads to inhibition of sympathetic activity, which decreases blood pressure (BP) and heart rate (HR), and results in sedation and pain control. (36,37) There is evidence that dexmedetomidine contributes to bleeding management. Although there is no evidence of its analgesic effect in orthognathic surgery, there is strong evidence in other procedures. It can be directly applied to the peripheral nervous system, causing a dose-dependent inhibition of C-fibres and α-fibres, and it acts on the locus coeruleus area, inhibiting nociceptive neurotransmission through the posterior horn of the spinal cord (37). Alpha-2 adrenergic receptors also act on the presynaptic membrane and inhibit the release of norepinephrine, which in turn induces hyperpolarization and inhibits pain signals to the brain. These drugs promote the release of acetylcholine from spinal interneurons, thereby increasing synthesis and releasing nitric oxide that regulates analgesia.

Hialotherapy, which is the application of cold compression through a facemask at a regulated temperature of 15 ºC, significantly reduces pain and oedema at 48-72 hours (12).

Postoperative pain management is usually achieved with NSAID, paracetamol, and corticoid combinations; opioids are rarely needed.

- Antibiotic prophylaxis.

Surgical site infection (SSI) is defined as an infection occurring within 30 days of surgery, or within 1 year in the case of patients receiving implants.

The risk of SSI is dependent on factors such as the duration of surgery, the wound class, and the patient´s American Society of Anaesthesiology (ASA) classification. Increased risk of surgical site infection is generally accepted as an indication for antibiotic prophylaxis (38).

Orthognathic surgery can be considered a clean contaminated surgery. These procedures would be expected to have higher infection rates than non-contaminated surgeries.

Our review of the literature identified the following recommendations.

1. Preoperative antibiotics reduce the risk of surgical infection (weak recommendation).

2. There is limited evidence supporting postoperative antibiotic dosing. A 3 - day regimen of postoperative antibiotics may reduce the risk of surgical site infection compared to 1 day (weak recommendation).

3. Further research is required in this area (38).

- DVT prophylaxis

Although deep venous thrombosis (DVT) increases the risk in surgical procedures,

it is uncommon in orthognathic surgery.

The risk of DVT is determined by patient characteristics and the clinical setting.

Prolonged periods in a head-up position can lead to venous pooling.

Further risk factors, as well as the use of hormone therapy (oral contraceptives or hormone replacement therapy) and obesity, are found in orthognathic patients.

Biochemical abnormalities (deficiencies of antithrombin, protein C or S,

and activated protein resistance [factor Leiden V mutation, etc]) can also predispose patients to DVT.

There are few published data on the incidence of DVT after oral and maxillofacial

surgery. One report, based on the recollections of 103 consultants, estimated the incidence at 0.00035%. Van de Perre *et al*. reported 3 episodes of deep vein

thrombosis in 2049 patients undergoing orthognathic surgeries, one of which resulted in pulmonary embolism ; however, these authors only measured parameters during the first 48 postoperative hours (39).

Even though orthognathic patients usually have few comorbidities, it is mandatory to minimise the risk of DVT, so we believe that mechanical thromboprophylaxis (compression stockings) should be used, as recommended in NICE guidelines (40).

- Emergence and extubation.

After completion of surgery and removal of the throat pack, the oropharynx should be suctioned to remove traces of blood, clots, and debris in order to avoid laryngospasm.

Positive pressure after extubation is also recommended.

Patients must be transferred to a PACU for the first few postoperative hours.

## Discussion

Optimizing the quality of care in orthognathic surgery will improve outcomes and speed up recovery. Careful anaesthesia management is directly involved in the result. We must know the procedure and the associated risks and protocolize the care and anticipate the potential problems.

This review emphasizes the importance of a multidisciplinary approach to anesthesia management in orthognathic surgery.

Effective airway management is crucial, especially in patients with anatomical challenges like syndromic patients. The use of videolaryngoscopy, particularly the McGrath videolaryngoscope, is highlighted for improving intubation success in difficult cases.

Fluid management is also critical in order to prevent postoperative edema. The use of intraoperative cooling and corticosteroids like dexamethasone is effective in reducing complications and improving recovery.

Intraoperative bleeding control, often managed through hypotensive anesthesia and tranexamic acid, is essential to facilitate the surgical procedure

Finally, the prevention of postoperative nausea and vomiting (PONV) is crucial for patient recovery. The review supports the use of total intravenous anesthesia (TIVA) and dexamethasone as effective strategies in reducing PONV.

In summary, successful anesthesia management in orthognathic surgery requires meticulous planning, advanced airway techniques, careful control of fluids and bleeding, and proactive prevention of postoperative complications.

## Conclusions

We could conclude in several items.

1.- Anaesthesia management in orthognathic surgery is critical due to the procedure´s complexity and the potential presence of syndromic features.

2.- Nasal intubation is performed to allow a clear view of the face and facial features during surgery; we might bear in mind the videolaryngoscopy to facilitate vocal cordal visualization in patients with difficulties in direct laryngoscopy.

3.-Critical aspects such as prevention of ocular injuries, the use of throat pack, deep venous prophylaxis, fluid therapy, edema management and infection prevention are addressed and are crucial for optimal recovery

4.-Multimodal analgesia is considered essential, combining local infiltration with opioid and non-opioids analgesia. Hialotherapy contributes to reducing postoperative pain and edema

Collaboration between anesthesiologists, surgeons, and other healthcare professionals is essential to optimize the quality of care in orthognathic surgery. Protocols and anticipation of potential issues are key aspects in achieving excellent outcomes.

In summary, comprehensive and careful anesthetic management, along with a multidisciplinary approach and the implementation of preventive protocols, is essential to improve outcomes and expedite recovery in orthognathic surgery.

## Figures and Tables

**Table 1:** Prophylactic doses and timing of administration of antiemetic drugs.

Drug group	Drug	Dose	Timing
Serotonin (receptor 5HT antagonists)	Ondansetron Granisetron Ramosetron Palonosetron	4-8 mg 1-2 mg iv 0,3 mg iv 0,075-0,25mg iv	End of Surgery
Corticosteroids	Dexamethasone	4-10 mg iv	After induction.
Butyrophenone	Droperidol Haloperidol	0,625-1,25 mg 1-2,5 mg	After induction. End of surgery.
Neurokinin antagonist (NK-1 receptors)	Fosaprepitant	150 mg iv	After induction.
Anticholinergics	Scopolamine	Transdermal patch 0.3/0.6 mg	Evening prior to surgery
Dopamine antagonists	Metoclopramide Amisulpride	10-25 mg iv 5-10 mg iv	End of surgery After induction
